# Isoliquiritigenin as a modulator of the Nrf2 signaling pathway: potential therapeutic implications

**DOI:** 10.3389/fphar.2024.1395735

**Published:** 2024-10-09

**Authors:** Mangmang Qiu, Kang Ma, Junfeng Zhang, Zhaohua Zhao, Shan Wang, Qing Wang, Hao Xu

**Affiliations:** ^1^ School of Basic Medical Sciences, Xi’an Medical University, Xi’an, China; ^2^ School of Basic Medicine, College of Medicine, Qingdao University, Qingdao, China; ^3^ Institute of Basic and Translational Medicine, Xi’an Medical University, Xi’an, China

**Keywords:** Nrf2, Isoliquiritigenin, disorders, anti-oxidant, natural compound

## Abstract

Nuclear factor erythroid-2-related factor 2 (Nrf2), a transcription factor responsible for cytoprotection, plays a crucial role in regulating the expression of numerous antioxidant genes, thereby reducing reactive oxygen species (ROS) levels and safeguarding cells against oxidative stress. Extensive research has demonstrated the involvement of Nrf2 in various diseases, prompting the exploration of Nrf2 activation as a potential therapeutic approach for a variety of diseases. Consequently, there has been a surge of interest in investigating the Nrf2 signaling pathway and developing compounds that can modulate its activity. Isoliquiritigenin (ISL) (PubChem CID:638278) exhibits a diverse range of pharmacological activities, including antioxidant, anticancer, and anti-tumor properties. Notably, its robust antioxidant activity has garnered significant attention. Furthermore, ISL has been found to possess therapeutic effects on various diseases, such as diabetes, cardiovascular diseases, kidney diseases, and cancer, through the activation of the Nrf2 pathway. This review aims to evaluate the potential of ISL in modulating the Nrf2 signaling pathway and summarize the role of ISL in diverse diseases prevention and treatment through modulating the Nrf2 signaling pathway.

## 1 Introduction

In recent decades, numerous studies established that oxidative stress contributes to a variety of human diseases, including cardiovascular disease, cancer, neurological disorder, and diabetes (Valko et al., 2007). Oxidative stress denotes the disruption in the equilibrium between oxygen free radicals and antioxidants (Bhimaraj and Tang, 2012). As part of normal physiological processes, ROS (such as superoxide radical, hydroxyl radicals, and hydrogen peroxide) are consistently produced within cells and are counteracted by the antioxidant defense system, thereby preserving a state of dynamic equilibrium and preventing harm to the body. Oxidative stress is caused by the overproduction of ROS, which can trigger the apoptosis of cells by damaging DNA, proteins, and lipids (Valko et al., 2007). Consequently, mitigating oxidative stress has the potential to be an effective therapeutic approach for a range of diseases.

The Nrf2 signaling pathway serves as the primary cellular antioxidative system in response to oxidative stress. As a transcription factor, Nrf2 regulates the expression of genes involved in antioxidant defense, thereby reducing ROS levels and safeguarding cells against oxidative damage. Due to its potent antioxidant effects, it has been reported that the Nrf2 signaling has been implicated in the pathogenesis of various diseases including cardiovascular diseases, brain diseases, kidney diseases, cancer et al. (Menegon et al., 2016; Tian et al., 2021; Liu Y. et al., 2022; He et al., 2024; Tang et al., 2024). Specifically, in the context of cardiovascular diseases, activation of Nrf2 has been demonstrated to confer protection against conditions such as myocardial ischemia-reperfusion injury and atherosclerosis (Tian et al., 2021; He et al., 2024). The study has shown that activation of Nrf2 can decrease infarct size and enhance cardiac function in myocardial ischemia-reperfusion injury, underscoring its therapeutic promise in cardiovascular pathologies (Tian et al., 2021). Similarly, in brain diseases such as Alzheimer’s and Parkinson’s, Nrf2 activation has been associated with neuroprotection (George et al., 2022). The activation of Nrf2 reduces the generation of ROS and improves mitochondrial function, thereby protecting neuronal cells from oxidative stress-induced damage (Brandes et al., 2021). In cancer, the role of Nrf2 is intricate and contingent upon the specific circumstances. While Nrf2 activation can protect normal cells from oxidative damage and reduce cancer risk, its persistent activation in cancer cells can confer resistance to chemotherapy and facilitate the proliferation of tumors. Therefore, the dual roles of Nrf2 inhibition should be taken into consideration in cancer therapy (Cleasby et al., 2014; Torrente et al., 2017; Pouremamali et al., 2022). Nrf2 also plays a crucial role in kidney diseases, where it helps to regulate genes that maintain homeostasis in the kidneys and mitigate oxidative stress and inflammation (Shelton et al., 2015; Liu Y. et al., 2022). Nrf2 has been shown to improve tubular injury and ameliorate kidney dysfunction, making it a promising target for kidney diseases management (Liu Y. et al., 2022). In summary, the Nrf2 signaling pathway is a master regulator of cellular-defense oxidative stress, with significant implications for a wide range of diseases. Moreover, there is presently a significant level of interest in the utilization of small molecular compounds to selectively target the Nrf2 signaling pathway as a means of treating a wide range of diseases (Zhu et al., 2020). Consequently, the quest for efficacious Nrf2 activators represents a promising novel therapeutic approach for the management of these ailments.

**FIGURE 1:**
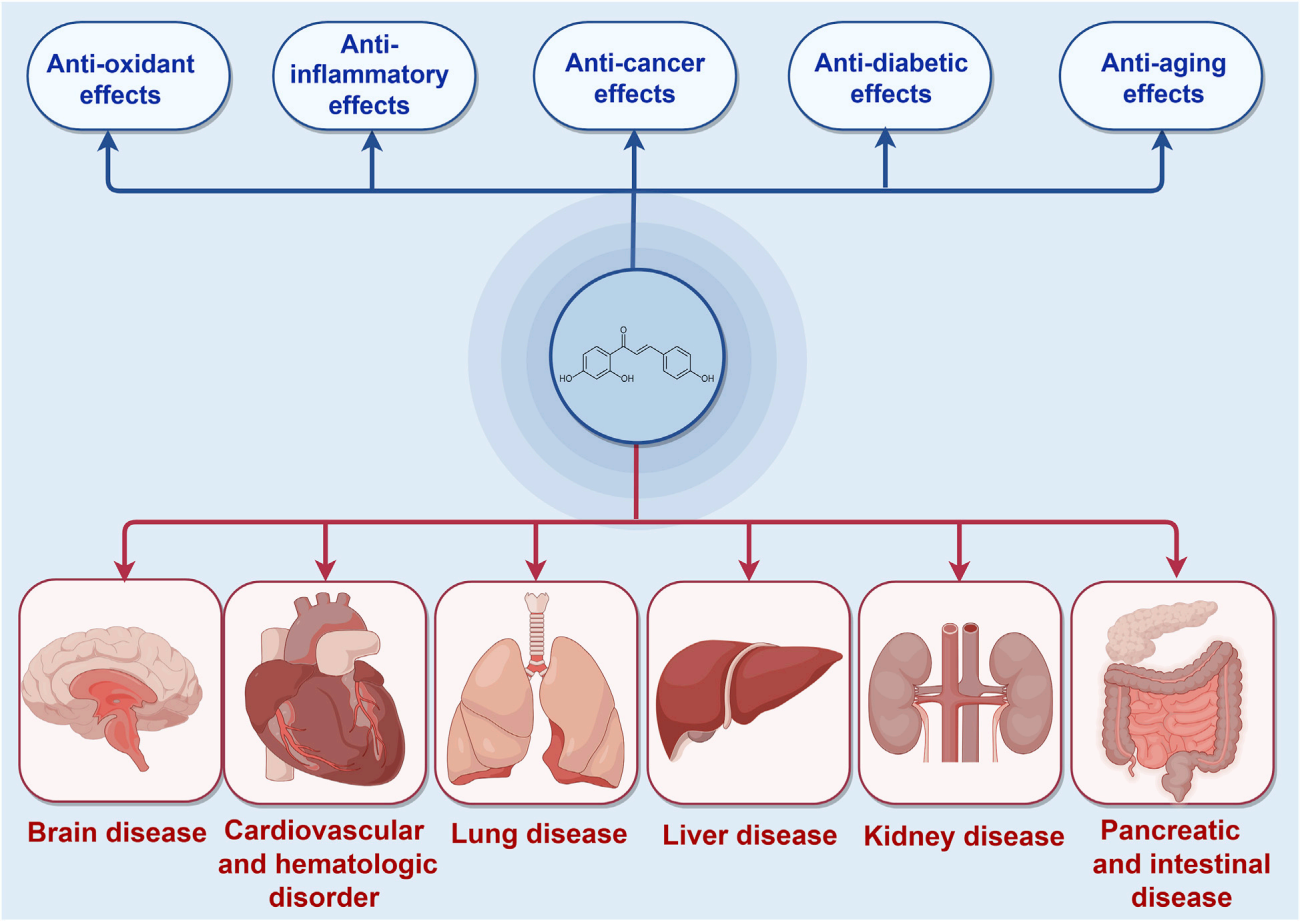
The protective effects of ISL in different diseases mediated by Nrf2 signaling pathway.

ISL, a chalcone-structured flavonoid derived from *Glycyrrhiza uralensis (licorice)* species (Li et al., 2010), has been found to possess therapeutic properties in various diseases including diabetes, cardiovascular diseases, kidney diseases, and cancer through the activation of the Nrf2 pathway (Xiong D. et al., 2018; Gu et al., 2020; Yao et al., 2022). ISL has been reported to exert an endogenous protective effect by facilitating Nrf2 nuclear translocation and modulating the expression of antioxidative enzymes such as nicotinamide adenine dinucleotide phosphate quinone oxidoreductase-1(NQO1), heme oxygenase-1 (HO-1), superoxide dismutase (SOD) et al. Consequently, the exploration of effective Nrf2 activators may present a novel potential therapeutic approach for the treatment of these diseases. Furthermore, ISL has been widely recognized as a safe phytochemical without any significant toxic, genotoxic, teratogenic properties in treating diseases (Zhao et al., 2019). Numerous studies have demonstrated the diverse pharmacological activities of ISL, including its antioxidant, anticancer, and anti-tumor properties (Iwata et al., 1999; Kwon et al., 2007; Aponte et al., 2008; Feldman et al., 2011; Chen Y. P. et al., 2013; Gaur et al., 2014; Zhao et al., 2019). These findings collectively suggest that ISL plays a pivotal role in the prevention and treatment of human diseases (Figure 1). In this context, we specifically explore the therapeutic potential of ISL in targeting the Nrf2 pathway and evaluate its underlying mechanisms, thereby providing valuable insights for future research endeavors in this field.

## 2 Structure and metabolism of ISL

**FIGURE 2:**
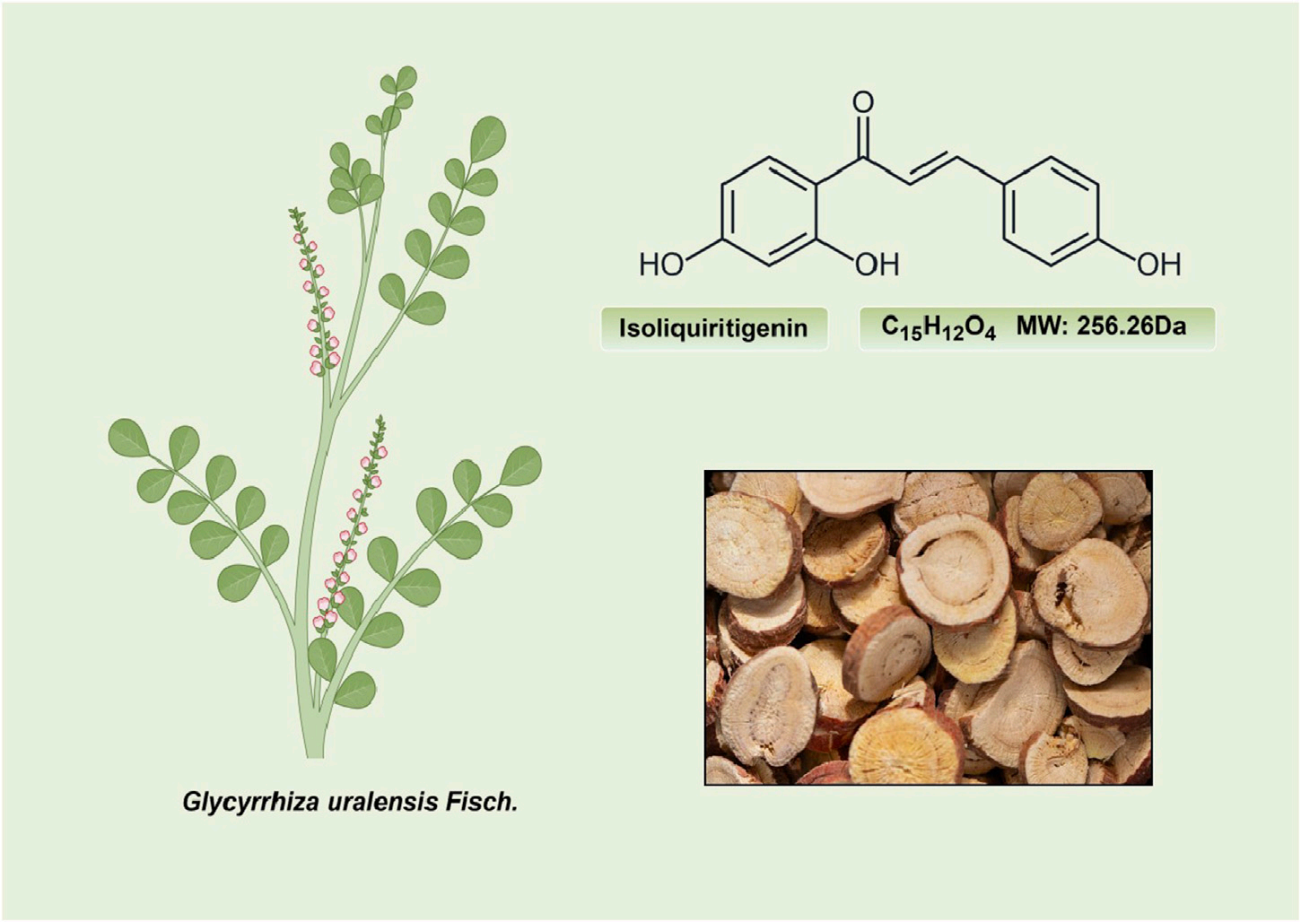
The structure and sources of ISL.

ISL (C_15_H_12_O_4_; MW: 256.26 Da) is a prominent member of the natural flavonoid compound group, primarily derived from the *licorice* root and various other plant species including *Cicer arietinum L* (Zhao et al., 2008)*, Radix Hedysari* (Zhao et al., 2008)*,* and *Crinum bulbispermum* (Ramadan et al., 2000). ISL (2′4′4-trihydroxychalcone) is classified within the chalcone family of flavonoids and finds application in the food additives and cosmetics industries (Cao et al., 2004; Wang Z. F. et al., 2020). The chemical structure of ISL is depicted in Figure 2. It exhibits limited solubility in water and manifests as a yellow crystalline solid (Wang Z. F. et al., 2020; Zhao et al., 2021). The research has determined that the bioavailability of ISL in rats following oral administration ranged from 22.70% to 33.62%, indicating a low level of oral bioavailability. ISL is absorbed in the intestine and is biotransformed through both phases II, in the liver after absorption (Guo et al., 2008). Numerous studies have demonstrated that ISL exhibits favorable uptake and elimination capabilities when administered through various routes, including intraperitoneal, intravenous, and oral inoculations (Han et al., 2011; Qiao et al., 2014). Moreover, it has been observed that orally ingested ISL is rapidly absorbed from the gastrointestinal tract. The presence of ISL in plasma can be detected within a 5-min timeframe, with its concentration peaking at 30 min. Following intraperitoneal injection of ISL, the liver exhibited the highest distribution of ISL, followed by the kidney, spleen, blood, lung, brain, and heart. The concentration of ISL in the blood reached its maximum level after 60 min (Han et al., 2011). Intravenous injection resulted in a rapid decrease in ISL concentration in plasma. The distribution half-life of ISL was found to be 0.3 h, while the elimination half-life for ISL doses of 10, 20, and 50 mg/kg were determined to be 4.9, 4.6, and 4.8 h, respectively (Qiao et al., 2014). Additionally, it was observed that intravenous administration of ISL resulted in its predominant distribution within highly vascularized tissues such as the heart, lungs, kidney, and liver. These findings suggest that the distribution pattern of ISL is influenced by the blood flow and perfusion of the respective organs (Qiao et al., 2014). Consequently, these results provide further substantiation for the potential therapeutic efficacy of ISL in treating disorders related to the cardiac, respiratory, and urinary systems. Furthermore, the significant accumulation of ISL in the kidney implies that it may serve as the primary organ for excretion of the compound. Despite its low expression in the brain due to high polarity (Li et al., 2008; Hua et al., 2010), it is intriguing to note that ISL can traverse the blood-brain barriers and exhibit neuroprotective effects in male MCAO-induced focal cerebral ischemic injury (Li et al., 2015). This could be relevant for the blood-brain barrier disruption after stroke (Wang et al., 2011). Presently, several approaches, including self-nanoemulsifying drug delivery systems (SNEDDS) and nanoparticles, are being explored to address the challenge of limited oral absorption and bioavailability associated with ISL.

## 3 Nrf2 structure and activation

**FIGURE 3:**
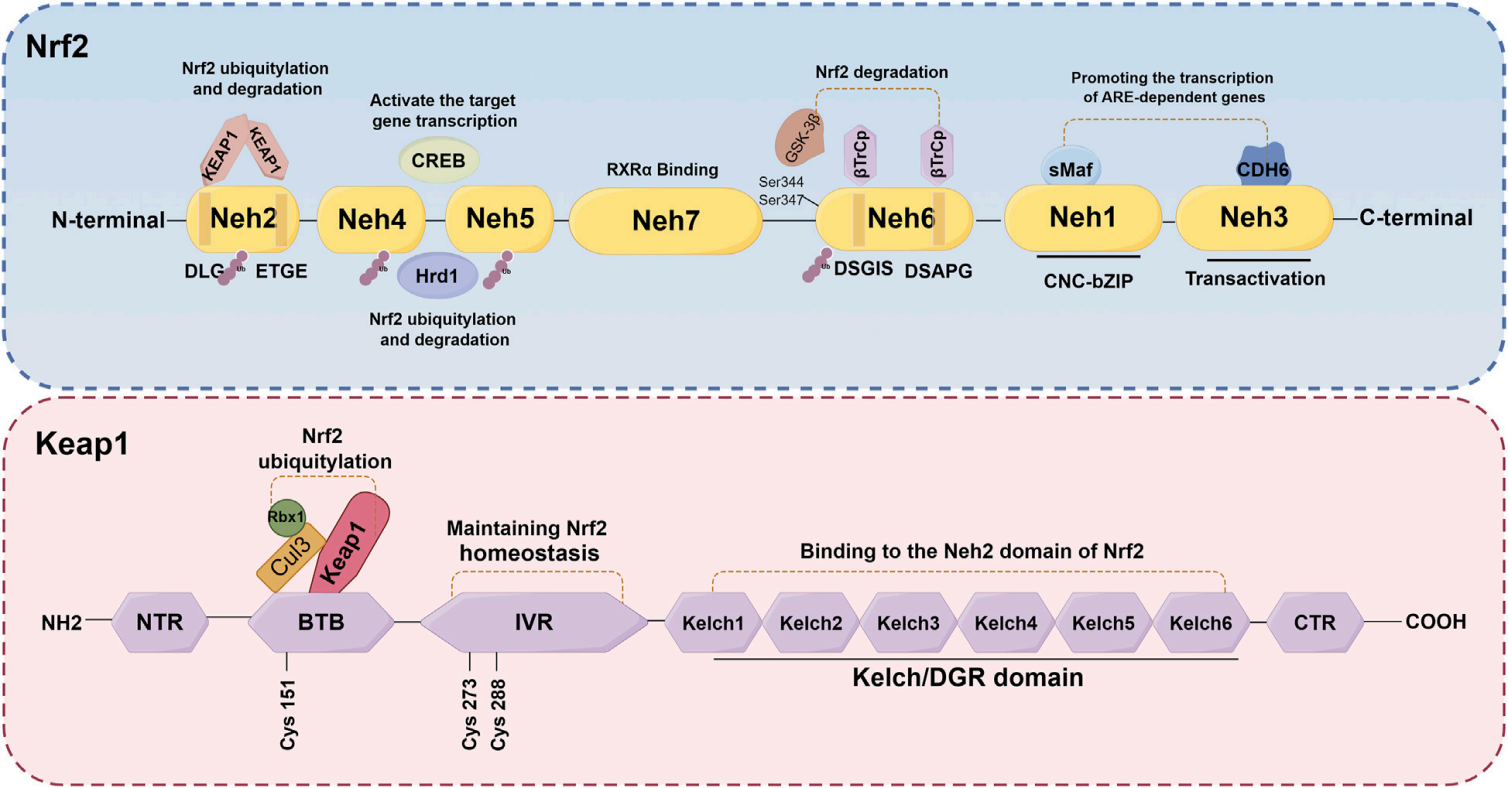
Schematic structures of Nrf2 and Keap1 protein. The protein Nrf2 is composed of seven distinct domains (Neh1-Neh7), each serving a specific function in regulating gene stability and transcriptional activity. The Keap1 protein consists of five distinct domains: NTR, IVR, BTB, DGR and CTR.

Nrf2, a crucial transcription factor involved in cellular defense mechanisms against oxidative stress, plays a significant role in maintaining equilibrium between free radicals and the body’s antioxidant system (O'Mealey et al., 2017). It is increasingly recognized as a pivotal regulator in various diseases, such as neurological diseases, cardiovascular diseases, lung diseases, and kidney disorders, among others (Chen et al., 2003; Pietsch et al., 2003; Chen et al., 2006; Rushworth et al., 2008; Kanninen et al., 2009). The *NFE2L2* gene encodes Nrf2, a transcription factor belonging to the cap 'n' collar subfamily of basic-region leucine zipper (bZIPs) (Chowdhry et al., 2013). Nrf2 has a molecular weight of about 100 kDa. The protein Nrf2 is composed of seven distinct domains (Neh1-Neh7), each serving a specific function in regulating gene stability and transcriptional activity (Goodfellow et al., 2020; Madden and Itzhaki, 2020; Shaw and Chattopadhyay, 2020) (Figure 3). Nrf2 contains three of these regions at its C-terminus: Neh1, Neh3, and Neh6. Neh1 contains a bZIP domain, which primarily interacts with small Maf proteins for dimerization (Madden and Itzhaki, 2020; Zhou et al., 2020). This interaction allows Nrf2 to recognize and bind to the antioxidant response element (ARE), thereby facilitating the transcription of relevant genes. Neh3, on the other hand, acts as the transactivation domain of Nrf2 and interacts with the transcriptional co-activator chromodomain helicase DNA binding protein 6, thereby facilitating the transcription of genes dependent on ARE and exhibiting antioxidant activity (Bellezza et al., 2018; Ooi et al., 2018). Besides, the Neh6 domain, which negatively regulates Nrf2 stability, is a serine-rich domain comprising two motifs (DSGIS and DSAPGS) that bind to β-transducin repeat-containing protein (β-TrCP) (Panieri et al., 2020). The Neh6 domain plays a role in the degradation of Nrf2 independent of Keap1. Glycogen synthase kinase-3β (GSK-3β) phosphorylates specific serine amino acid residues (Ser344 and Ser347) within the Neh6 domain, thereby facilitating the ubiquitination and subsequent proteasomal degradation of the Nrf2 protein (Zhang et al., 2020). Conversely, GSK-3β activity is suppressed when it is phosphorylated on Ser21/9 residue, which results in activating Nrf2 and promotes the transcription of its downstream antioxidant and increase the antioxidant capacity (Hayes et al., 2015). The Neh4 and Neh5 domains, situated in the N-terminal halves, are responsible for the transcriptional regulation of downstream target genes. Upon translocation of Nrf2 to the nucleus, only the Neh4 and Neh5 domains interact with cAMP response element-binding protein (CREB) to activate transcription of the target genes (Bellezza et al., 2018). Furthermore, scholars have also discovered that the Neh4/Neh5 domains engage in interactions with the E3 ubiquitin ligase, hydroxy methylglutaryl coenzyme A reductase degradation 1, thereby facilitating the ubiquitylation and subsequent degradation of Nrf2(Wu et al., 2014). The Neh2 domain, which serves as a negative regulatory region and the primary binding site for Keap1, primarily comprises DLG and ETGE motifs (Chowdhry et al., 2013). Elucidating the underlying mechanisms, it has been found that the homodimer double glycine repeat domain (DGR) of Keap1 binds to the DLG and ETGE motifs of Nrf2, leading to the ubiquitination of lysine residues in Nrf2. Consequently, this process results in the inactivation and degradation of Nrf2(Zenkov et al., 2017; Bellezza et al., 2018; Tonelli et al., 2018). Additionally, the investigation revealed that the Neh7 domain engages in an interaction with retinoic X receptor α, resulting in the repression of Nrf2 activity and the transcription of its target genes (Wang et al., 2013). Generally, the activity of Nrf2 is governed by the regulated maintenance of Nrf2 protein levels, which is achieved through both Keap1-dependent and Keap1-independent mechanisms. The subsequent sections provide a comprehensive overview of these two prominent regulatory mechanisms.

### 3.1 Keap1-dependent regulation

**FIGURE 4:**
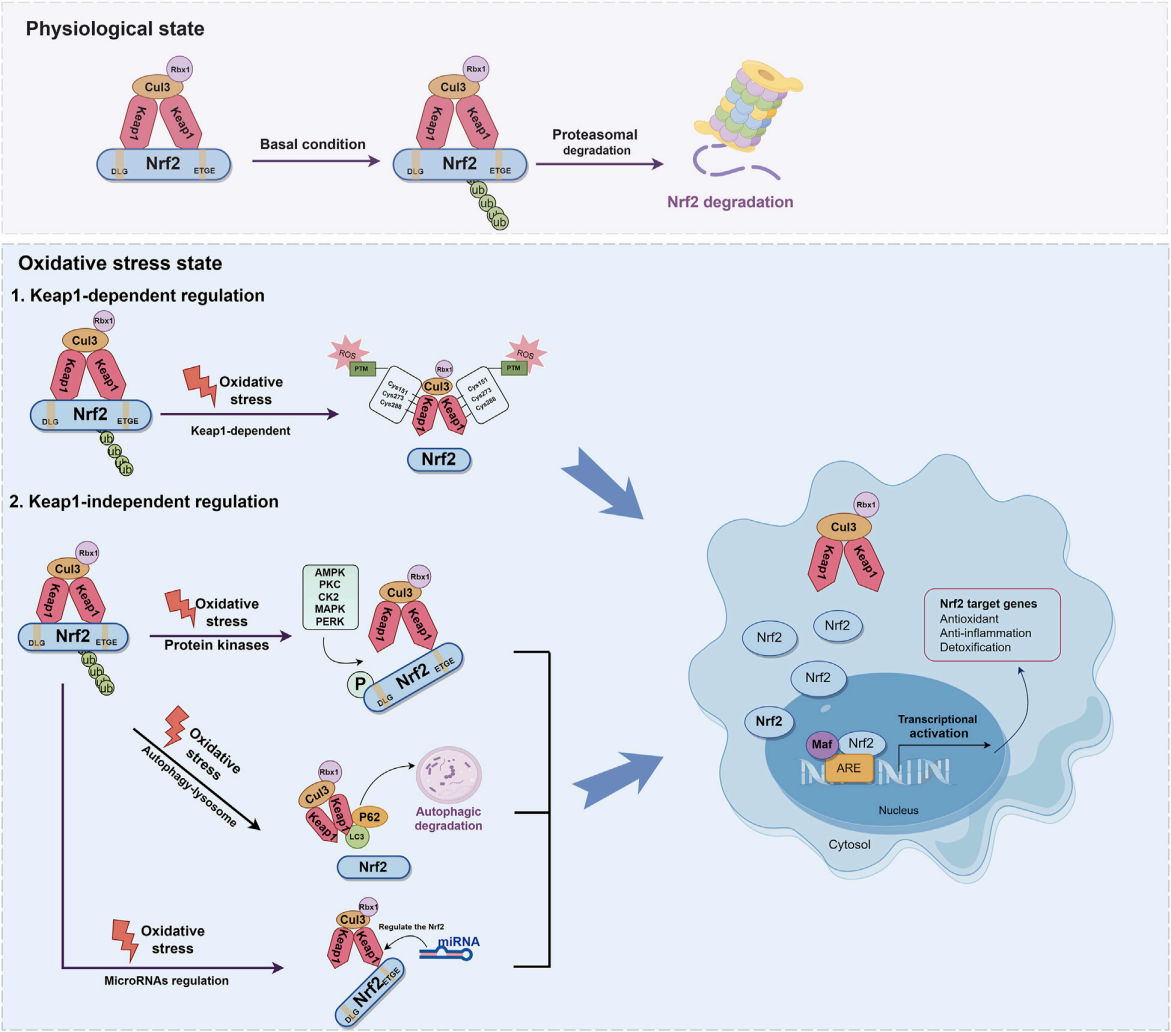
Molecular mechanisms of Keap1-dependent and Keap1-independent modulation of Nrf2. The regulation of Nrf2 involves both the Keap1-dependent and Keap1-independent pathways. Under normal physiological conditions, dimers of Keap1 exist in the cytoplasm and are substrate adapters protein for Cul3 E3 ubiquitin ligases. Nrf2 is ubiquitinated by E3 ubiquitin ligase, and degraded by the 26S proteasome. During oxidative stress, ROS can modify the reactive cysteine residues within Keap1, such as Cys151 in the BTB domain and Cys273, Cys288 in the IVR domain. This interaction disrupt the Keap1-Cul3-Rbx1 ubiquitin ligase complex and Nrf2 interaction, leading to the dissociation of Nrf2 from Keap1. When Nrf2 dissociates from Keap1, it translocates into the nucleus where it forms heterodimers with Maf proteins within the Neh1 domain. In promoter regions, Nrf2 forms a complex with the ARE, thereby enhancing the antioxidant capacity by facilitating transcription of downstream antioxidant genes. Nrf2 can also be activated via the autophagy-lysosome pathway. The protein p62 serves as a selective autophagy receptor, facilitating the targeting of ubiquitinated substrates to autophagosomes for degradation. This interaction facilitates the integration of Keap1 into the autophagosome for degradation through the autophagy pathway, resulting in the separation of Nrf2 and Keap1 and thereby promoting the expression of antioxidant enzyme genes. miRNA plays a key role in the regulation of Nrf2 and contributes to its antioxidant capacity.

It is widely recognized that the regulation of Nrf2 involves both the Keap1-dependent and Keap1-independent pathways. The Keap1-dependent mechanism serves as the primary pathway for regulating Nrf2. In normal cells, Keap1 acts as a principal negative regulator of Nrf2 by ubiquitinating and degrading it. This negative regulation effectively controls Nrf2 activity and tightly regulates its concentration (Baird and Yamamoto, 2020; Kopacz et al., 2020). The relative molecular weight of Keap1 is 69KD (Canning et al., 2013). The Keap1 protein consists of five distinct domains: the N-terminal domain (NTR), the intervening region (IVR), the Broad complex, Tramtrack and Bric-à-Brac domain (BTB), the DGR, and the C-terminal region (CTR). Among these domains, the DGR domain, also referred to as the Kelch domain, plays a crucial role in the binding of Keap1 to the Neh2 domain of Nrf2(Ooi et al., 2018; Zhou et al., 2020) (Figure 3). Under normal physiological conditions, dimers of Keap1 exist in the cytoplasm and are substrate adapters protein for cullin3 (Cul3) E3 ubiquitin ligases (Suzuki et al., 2019). The additional study has also revealed that the presence of seven lysine residues positioned between the DLG and ETGE motifs in the Neh2 domain renders them susceptible to poly-ubiquitination, thereby facilitating the degradation of Nrf2 by the 26S proteasome system (Shibata et al., 2008; Harder et al., 2015; Mohan and Gupta, 2018) (Figure 4). Furthermore, the IVR domain in Keap1, particularly Cys273 and Cys288, assumes a crucial role in maintaining the stability of the Nrf2-Keap1 complex and ensuring Nrf2 homeostasis. Consequently, in the absence of external stressors, Nrf2 undergoes continuous synthesis and degradation, resulting in minimal net accumulation (Wakabayashi et al., 2004). Notably, Nrf2 exhibits a remarkably short half-life of approximately 10–30 min (Nguyen et al., 2003; Stewart et al., 2003). In contrast, the Cul3-Keap1-E3 ligase’s capacity to ubiquitinate Nrf2 is hindered during periods of stress, causing Nrf2 to relocate to the nucleus and manifest its antioxidative properties. During oxidative stress, it is important to mention that ROS or electrophiles have the ability to cause changes in the structure of the Keap1 domain. Specifically, ROS can modify the reactive cysteine residues within Keap1, such as Cys151 in the BTB domain and Cys273, Cys288 in the IVR domain (Tebay et al., 2015) (Figure 4). This interaction has the potential to disrupt the Keap1/Cul3/RING-box protein 1 (Rbx1) ubiquitin ligase complex and Nrf2 interaction, leading to the dissociation of Nrf2 from Keap1 and subsequent suppression of ubiquitin-dependent degradation of Nrf2. When Nrf2 dissociates from Keap1, it translocates into the nucleus where it forms heterodimers with Maf proteins within the Neh1 domain and binds to the coactivator CREB protein, thereby enabling Nrf2 to possess DNA binding capability. In promoter regions, Nrf2 forms a complex with the ARE, thereby enhancing the antioxidant capacity by facilitating transcription of downstream antioxidant genes. Notable examples of these antioxidant genes encompass NQO1, glutathione S-transferase (GST), HO-1, and glutamate-cysteine ligase modifier subunit (GCLM) (Itoh et al., 1997; Buendia et al., 2016; Tonelli et al., 2018) (Figure 4).

### 3.2 Keap1-independent regulation

In addition to the Keap1-dependent Nrf2 regulation pathway, Nrf2 activity is subject to stringent regulation through various mechanisms (Kaspar et al., 2009; Ichimura et al., 2013; Huang et al., 2014; Lin, 2019). Recent research has demonstrated that Nrf2 can also be activated via the autophagy-lysosome pathway, which is a highly regulated process responsible for the removal of damaged proteins and organelles (Ichimura et al., 2013; Zhang L. et al., 2016). The protein sequestosome 1 (SQSTM1/p62) serves as a selective autophagy receptor, facilitating the targeting of ubiquitinated substrates to autophagosomes for degradation. As an adaptor protein, p62 plays a critical role in connecting autophagy with the Keap1-Nrf2 signaling pathway (Mizushima and Hara, 2006). This interaction facilitates the integration of Keap1 into the autophagosome for degradation through the autophagy pathway, resulting in the separation of Nrf2 and Keap1. This facilitates the movement of Nrf2 into the nucleus, thereby promoting the expression of antioxidant enzyme genes (Kageyama et al., 2018). Additionally, p62 is identified as one of the genes regulated by transcription. Nrf2 can activate and induce the production of p62, subsequently reactivating Nrf2, thus establishing a positive feedback loop (Santarino et al., 2017) (Figure 4).

The amino acid sequence of Nrf2 also provides multiple phosphorylation sites for protein kinases, such as serine, threonine, and tyrosine residues, which play a role in Nrf2 regulation. It has been shown that these modifications to Nrf2 may lead to its degradation, nuclear translocation, and nuclear export (Huang et al., 2002; Cullinan and Diehl, 2004; Keum et al., 2006; Pi et al., 2007). The phosphorylation of Neh2 by protein kinase C (PKC) results in the release of Nrf2 from Keap1 and subsequent enhancement of transcriptional activity of Nrf2. Further supporting the notion that PKC directly phosphorylates Ser40 within Neh2 domain is this finding (Huang et al., 2002). Additionally, it has been reported that GSK-3β phosphorylates the Nrf2 Neh6 domain (DSGIS domain) through β-TrCP, thereby inhibiting Nrf2 activity and exerting a negative regulatory effect (Hayes et al., 2015). The specific mechanism has been reported that β-TrCP has the ability to recognize the phosphorylated DSGIS motif in the Neh6 domain, leading to the formation of the SKP1-CUL1-F-box protein (SCF)β-TrCP E3 ubiquitin ligase complex. Ultimately, this complex is involved in Nrf2 ubiquitination and degradation (Latres et al., 1999; Zheng et al., 2002; Hayes et al., 2015). Additionally, it has been reported that casein kinase two is capable of phosphorylating the Neh4 and Neh5 domains of Nrf2(Pi et al., 2007; Apopa et al., 2008). On the other hand, protein kinase RNA-like endoplasmic reticulum kinase (PERK) directly phosphorylates Nrf2, enhancing Nrf2-Keap1 dissociation and Nrf2 antioxidant pathways (Cullinan et al., 2003). Furthermore, PERK also facilitates the upregulation of the bZIP transcription factor activating transcription factor 4, which interacts with Nrf2 and triggers the activation of Nrf2 targeted genes (He et al., 2001; Ma et al., 2018). The mitogen-activated protein kinase (MAPK) pathway, known for its involvement in kinase signaling cascades, has been demonstrated that it regulates Nrf2 activity. However, the relationship between MAPK and Nrf2 appears contradictory. For instance, there is evidence that p38 MAPK can both positively and negatively modulate the antioxidant activity of Nrf2(Alam et al., 2000; Yu et al., 2000; Keum et al., 2006; Kocanova et al., 2007; Tsai et al., 2011). On the other hand, extracellular signal-regulated kinase (ERK) and c-Jun N-terminal kinase (JNK) are more likely to positively regulate Nrf2 activity, as depicted in Figure 4(Keum et al., 2003; Nguyen et al., 2003; Xu et al., 2006; Varì et al., 2011; Choi et al., 2016; Feng et al., 2018).

Moreover, numerous studies have shed light on the correlation between Nrf2 and microRNA (miRNA) (Stachurska et al., 2013; Alural et al., 2015; Yang et al., 2021). miRNA, a crucial type of non-coding single-stranded RNA responsible for gene expression regulation, plays a role in the regulation of Nrf2, with approximately 85 miRNAs potentially involved (Bartel, 2004; Papp et al., 2012). Experimental evidence has previously confirmed that miR-1225-5p directly interacts with Keap1, leading to increased Nrf2 levels and subsequent translocation of Nrf2 into the nucleus, resulting in upregulated HO-1 levels (Yang et al., 2021). Additionally, there are reported indications that miR-132 and miR-34a may modulate Nrf2 mRNA expression based on miRNA expression profiles (Stachurska et al., 2013; Alural et al., 2015). The role of miR-141-3p in enhancing the stability of Nrf2 by targeting Keap1 has been demonstrated (van Jaarsveld et al., 2013; Shi et al., 2015; Xu et al., 2021). Overall, Nrf2 is regulated by miRNA as depicted in Figure 4.

## 4 The role of ISL in targeting different diseases via Nrf2 pathway

### 4.1 Brain diseases

**FIGURE 5:**
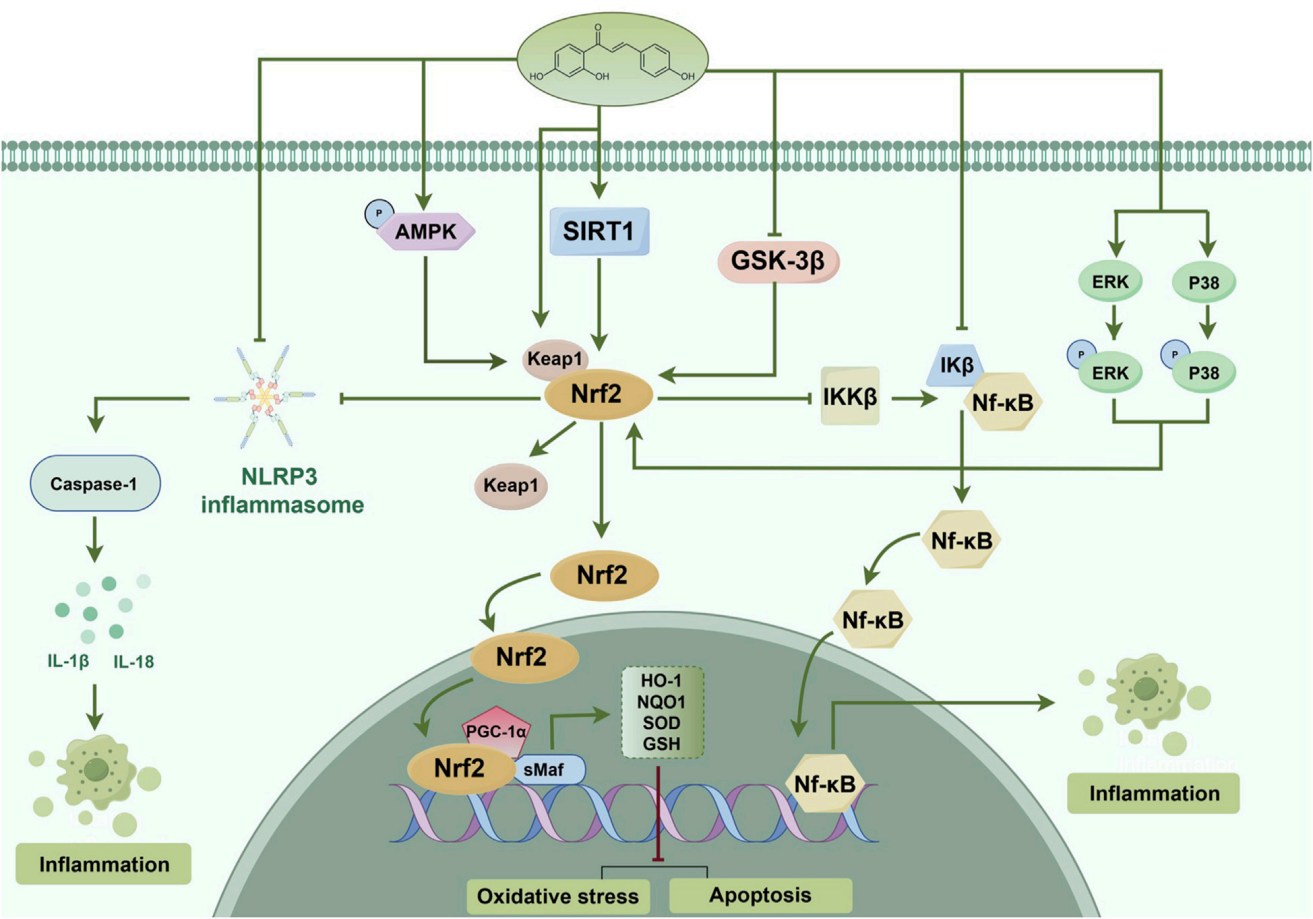
Probable mechanism of action of ISL on the Nrf2 pathway. ISL alleviated oxidative stress, cell apoptosis and inflammation via activating the AMPK/Nrf2 antioxidant signal pathway and inhibiting the NLRP3 inflammasome activation and NF-κB pathway. The inhibitory effect of ISL on NLRP3 inflammasome and NF-κB pathway may be through both Nrf2 and non-Nrf2-independent signaling events. ISL also phosphorylates MAPKs and upregulates the Keap1, which induces the separation of Nrf2 from Keap1, activation of Nrf2 signaling, and enhancement of detoxification phase II enzymes activity. ISL activates SIRT1 to promote the Nrf2 antioxidant signaling pathway. Moreover, ISL also increase its antioxidant ability and decrease in inflammatory, cell apoptosis through activating the PGC-1α/Nrf2 pathway. Besides, ISL also can inhibit GSK-3β activity by phosphorylation to enhance the Nrf2 related antioxidant gene expression and inhibit the NF-κB induced inflammatory gene. (↑) represent positive effect while (⊥) represent negative effect on the pathway.

ISL is recognized as an effective antioxidant and cerebroprotective through the Nrf2/ARE signaling pathway (Foresti et al., 2013; Zeng et al., 2017; Zhu et al., 2019b). The investigation has revealed that ISL effectively mitigated early brain injury induced by subarachnoid hemorrhage, as evidenced by its ability to inhibit neuronal apoptosis, oxidative stress, and brain edema. Moreover, the administration of ISL augmented the expression of Nrf2, while the inhibition of Nrf2 activity counteracted the antioxidant and neuroprotective effects of ISL (Liu J. Q. et al., 2022). Another study has demonstrated that ISL significantly ameliorated neurological deficits in patients diagnosed with Parkinson’s disease (PD). The underlying mechanism may be attributed to the attenuation of neuro-inflammation through the Nrf2/ARE pathway. The researchers observed that the administration of ISL at a dosage of 20 mg/kg effectively reduced the rotational behavior in PD mice, enhanced the expression of tyrosine hydroxylase, and decreased the expression of α-synuclein. Additionally, ISL not only decreased the expression of ionized calcium bindingadaptor molecule-1 (Iba-1), interleukin-1β (IL-1β), and tumor necrosis factor-α (TNF-α), but also upregulated the expression of Nrf2 and NQO1 (Huang et al., 2022). In the lipopolysaccharide (LPS)-induced cognitive impairment rat model, ISL also prevented neuronal damage and cognitive impairment by maintaining antioxidant ability and suppressing neuroinflammation. The protective effects of ISL have been shown to be exerted through inhibiting GSK-3β activity through increasing the expression levels of phosphorylated (p)-GSK-3β, thereby disinhibiting Nrf2 and upregulating the gene expression of Nrf2-controlled anti-oxidant genes. Besides, ISL also reduced pro-inflammatory cytokine production (Zhu et al., 2019a). The antioxidant mechanism of ISL is associated with reducing vascular permeability, enhancing neurological functions, and mitigating neuronal apoptosis in order to alleviate traumatic brain injury (TBI). This is achieved through the promotion of nuclear translocation of Nrf2 and the activation of the Nrf2-regulated ARE (Zhang et al., 2018a) (Figure 5). Supplementary Table 1 provides an overview of the Nrf2-related therapeutic effects of ISL in brain injury.

### 4.2 Liver diseases

The prevalence of liver disease has increased significantly in recent years (Zhang et al., 2015). One significant factor contributing to acute liver dysfunction is drug-induced liver toxicity (Rabinowich and Shibolet, 2015). Doxorubicin, a widely utilized cancer chemotherapeutic agent, is commonly employed in the treatment of various tumor types, such as breast cancer, lymphomas, and leukemia, among others (Renu et al., 2019). However, the administration of doxorubicin is associated with adverse effects including cardiotoxicity, hepatotoxicity, and nephrotoxicity (Pugazhendhi et al., 2018). Given that the liver serves as a crucial metabolic organ in the human body, it has been observed that during doxorubicin therapy, the liver is exposed to high concentrations of the drug and experiences the most pronounced impact (Carvalho et al., 2009). Research has demonstrated that doxorubicin-induced hepatotoxicity is a complex process involving multiple molecules, primarily driven by oxidative stress, inflammation, and apoptosis (Prasanna et al., 2020). There is substantial evidence that doxorubicin inhibits the expression of Nrf2 and reduces the expression of antioxidant enzyme-related genes, thereby intensifying the oxidative stress damage caused by doxorubicin in the liver. As evidenced by the downregulation of serum aminotransferase levels following ISL administration, ISL may mitigate doxorubicin-induced hepatotoxicity. This protective effect is achieved through the induction of Nrf2-mediated antioxidant signaling and the inhibition of nuclear factor kappa-B (NF-κB) activity (Al-Qahtani et al., 2022). Furthermore, it has been observed that emodin, the primary constituent of *Cassia obtusifolia, Aloe vera,* and *Polygonum multiflorum,* can potentially induce hepatotoxicity, especially when administered in high doses and over an extended period of time (Wang et al., 2009; Panigrahi et al., 2015; Dong et al., 2016). It has been demonstrated that emodin exerts its hepatotoxic effects by generating ROS and activating mitochondria-dependent pathways (Dong et al., 2018). Additionally, another study has revealed that the combination of ISL and emodin exhibits a significant hepatoprotective effect by enhancing cellular activity, reducing the production of ROS, and augmenting antioxidant capacity. Additionally, ISL has been found to promote the separation of Keap1 from Nrf2, resulting in the translocation of Nrf2 into the nucleus and subsequent induction of phase-II detoxifying enzyme expression (Ni et al., 2022). Furthermore, ISL has demonstrated significant mitigation of triptolide-induced acute hepatotoxicity by reducing hepatic oxidative stress and the accumulation of both endogenous bile acids and exogenous triptolide and its metabolites (Hou et al., 2018). It has been shown that ISL exerts its protective effects by enhancing Nrf2 expression, its downstream NQO1 expression, and hepatic influx and efflux transporters expression (Hou et al., 2018). As previously mentioned, ISL has demonstrated its potential as a hepatoprotectant and as a means to mitigate the detrimental impacts of other substances on liver function. Additionally, according to studies, ISL protects against acute liver failure caused by LPS/D-galactosamine (LPS/D-GalN), with the extent of this protection being dependent on the dosage administered. This protective mechanism is believed to be mediated through the peroxisome proliferator-activated receptor-gamma coactivator (PGC-1α)/Nrf2 pathway (Wang et al., 2021) (Figure 5). The therapeutic effects of ISL in liver diseases, specifically those related to Nrf2, are summarized in Supplementary Table 1.

### 4.3 Pancreatic and intestinal diseases

Severe acute pancreatitis (SAP) is a highly prevalent and fatal digestive disorder characterized by an abnormal inflammatory process affecting the pancreas (Lankisch et al., 2015; Lu et al., 2016). The occurrence of severe intestinal dysfunction is closely linked to acute pancreatitis (Karakayali, 2014). It has been suggested that mitigating SAP complications, including intestinal dysfunction, through appropriate interventions can be beneficial (Xiong Y. et al., 2018). Consequently, ameliorating bowel dysfunction may contribute to the improvement of SAP-related injuries. A prior investigation has demonstrated that the restoration of the impaired intestinal barrier is facilitated by the Nrf2/ARE signaling pathway (Hu et al., 2018). In a recent study conducted by Zhang et al., it was discovered that mice lacking Nrf2 exhibited heightened vulnerability to injury in the context of SAP. This finding elucidates the crucial reparative function of Nrf2 in maintaining the intestinal barrier intact. Furthermore, the researchers also validated the significant ameliorative effects of ISL on both pancreatic and intestinal damage, as well as its ability to restore intestinal function. These effects were mediated through the Nrf2/NF-κB pathway, which was found to mitigate oxidative stress and inflammation in the animal model of SAP (Zhang et al., 2018b). Furthermore, an additional study has demonstrated that ISL displays substantial antioxidant activity and mitigates pancreatic histopathological damage in a dose-dependent manner by diminishing the levels of serum amylase and lipase. Additionally, ISL has the ability to enhance the Nrf2/HO-1 signal pathway’s protein expression. There is evidence that the ML385 (Nrf2 inhibitor) and the zinc protoporphyrin (HO-1 inhibitor) significantly counteract the protective effects of ISL. Collectively, the aforementioned data provide novel evidence that implicates ISL as a protective agent in the experimental model of SAP induced by L-arginine, primarily through its ability to alleviate oxidative stress (Liu et al., 2018). Furthermore, ISL has demonstrated efficacy in preventing inflammatory bowel disease, with its protective effects potentially mediated by activating Nrf2 and its downstream targets (Chi et al., 2017). Consequently, these findings substantiate the potential of ISL as a viable therapeutic option for the clinical management of SAP (Figure 5). Supplementary Table 1 presents a comprehensive overview of the therapeutic effects of ISL on Nrf2-related pathways in both pancreatic and intestinal disorders.

### 4.4 Cardiovascular diseases and hematologic disorder

Globally, cardiovascular disease remains the leading cause of morbidity and mortality (Sidney et al., 2016). Oxidative stress represents a significant mechanism implicated in the occurrence of acute myocardial infarction (AMI). In a study utilizing an animal model of AMI, specifically the ligation of the left anterior descending coronary artery, researchers observed that ISL exhibited a remarkable ability to reduce myocardial infarct size and enhance the recovery of cardiac function. The mechanism involved the Nrf2/HO-1 pathway activation, which resulted in the mitigation of myocardial inflammation and oxidative stress in mice with AMI(Yao et al., 2022). Additionally, there is a strong association between diabetes and cardiovascular diseases (Radovits et al., 2009). Pathological changes, including the presence of atheroma plaque, were observed in the aorta of the diabetic group. However, administration of ISL (20 mg/kg) for 8 weeks significantly suppressed endothelin-1 expression in the aortic endothelium of rats with streptozotocin-induced diabetes and preserved the structural integrity of the aorta. It is noteworthy to mention that ISL has the potential to enhance redox homeostasis in the aorta and prevent the apoptosis of endothelial cells through the Nrf2/cysteinyl aspartate specific proteinase-3 (Caspase-3) pathway (Alzahrani et al., 2021). Additionally, another study demonstrated that ISL can inhibit hypertrophy, fibrosis, and apoptosis in H9c2 cells induced by high glucose, both *in vitro* and *in vivo*, by suppressing inflammation and oxidative stress. Furthermore, the significant protective effect of ISL was attributed to the inhibition of MAPKs and the induction of the Nrf2 signaling pathway. These aforementioned studies collectively indicate that ISL holds promise for the development of therapeutic interventions against diabetic cardiomyopathy (Gu et al., 2020) (Figure 5). Supplementary Table 1 shows Nrf2-related therapeutic effects of ISL in cardiovascular disease and hematologic disorder.

### 4.5 Lung diseases

Acute lung injury (ALI)/acute respiratory distress syndrome is a significant clinical syndrome associated with diffuse inflammation and respiratory failure, resulting in high mortality rates and limited therapeutic interventions (Ware and Matthay, 2000; Baetz et al., 2005; Sarma and Ward, 2011). Previous research has demonstrated that ISL exhibits potent antioxidant properties and has the potential to mitigate lung injury (Liu et al., 2017). To investigate this further, the researchers conducted *in vitro* studies using a tert-Butyl hydroperoxide (t-BHP)-induced RAW.264.7 injury model, which mimics lung injury. The findings revealed that treatment with ISL effectively reduced the ROS production and attenuated cellular toxicity in RAW.264.7 cells. *In vivo* experiments have demonstrated the protective effect of ISL in reducing LPS-induced lung injury in mice with ALI. The role of ISL is primarily emphasized through its impact on lung histopathology, reduction of lung edema, and prevention of protein leakage. Additionally, ISL has been found to inhibit the production of ROS, myeloperoxidase (MPO), and malondialdehyde (MDA). Furthermore, ISL significantly improves the LPS-induced decrease in glutathione (GSH) and SOD levels *in vivo*. According to the study, activation of the adenosine 5′-monophosphate (AMP)-activated protein kinase (AMPK)/Nrf2/ARE signaling pathway is crucial to effectively protecting against lung injury (Liu et al., 2017). Additionally, a separate study has demonstrated the notable protective effects of ISL against chronic obstructive pulmonary disease (COPD) induced by cigarette smoke (CS) (Yu et al., 2018). COPD is a highly prevalent disease, with CS being widely recognized as its primary risk factor (Du et al., 2017; Vogelmeier et al., 2017). Prolonged exposure to CS leads to chronic inflammation and oxidative stress, resulting in significant impairment of lung function (Chen et al., 2010; Du et al., 2017). In the aforementioned study, it was observed that ISL effectively mitigated lung pathological injuries induced by CS, as evidenced by the reversal of wet/dry ratio and MPO activity. This notable effect can be attributed to a significant reduction in inflammatory cell infiltration, improvement in the redox state, and modulation of the Nrf2 signal pathway (Yu et al., 2018) (Figure 5). In Supplementary Table 1, we provide an overview of the therapeutic effects of ISL on Nrf2-related mechanisms in lung diseases.

### 4.6 Kidney diseases

Hypertensive renal injury is a prominent risk factor for renal injury, playing a significant role in developing of end-stage nephropathy and the requirement for dialysis (Dugbartey, 2017). A study conducted on hypertensive renal injury has initially demonstrated that ISL can mitigate the inflammatory cytokine’s production, apoptosis induced by oxidative stress, and excessive deposition of extracellular matrix. Additionally, it has been demonstrated that ISL can effectively safeguard against renal injury by activating the Nrf2 and NF-κB pathways. These findings indicate the potential preventive and therapeutic benefits of ISL in the context of hypertensive renal injury (Xiong D. et al., 2018) (Figure 5). The therapeutic effects of ISL on kidney diseases, particularly those related to Nrf2, are summarized in Supplementary Table 1.

## 5 ISL nanoformulations and ISL metabolites

ISL exhibits high solubility in organic solvents but low solubility in water (Xie et al., 2019). ISL has the low bioavailability *in vivo* due to factors such as its poor solubility, high first-pass metabolism and rapid excretion (Zhang et al., 2013; Qiao et al., 2014). Notably, the term inadequate bioavailability suggests that the desired effects under treatment of ISL are difficult to achieve, thereby hinders its clinical application. To increase the dissolution and improve the oral bioavailability of ISL, encapsulated ISL nanoparticles or nano-ISL have been developed. Next, We have summarized various ISL nanocarriers in preclinical studies and their potential applications in Table 1.

**TABLE 1:** Summary of the most relevant preclinical studies evaluating the ISL nanoformulations.

Nano-formulation	Particle size (nm)	Disease	Effects	References
ISL self-microemulsifying drug delivery system (SMEDDS)	44.78 ± 0.35	Hyperuricemia	ISL-SMEDDS can improve the solubility and oral bioavailability of ISL, and exert the anti-hyperuricemic activity.	Zhang et al. (2019a)
ISL-loaded F127/P123 polymeric micelles (ISL-FPM)	20.12 ± 0.72	—	ISL-FPM enhanced solubility, bioavailability and antioxidant activity of ISL.	Xie et al. (2019)
ISL loaded zein/caseinate nanocomplexes (ISL-NPs)	137.32 ± 2.54	Ulcerative colitis	ISL-NPs can have stronger drug-loading capacity, increase the retention of ISL in colonic inflammation sites and effectively alleviate ulcerative colitis symptoms.	Xiao et al. (2022)
ISL self-nano-emulsifying drug delivery system (ISL-SNEDDS)	33.4 ± 2.46	Eosinophilic esophagitis	ISL-SNEDDS can improve the bioavailability of ISL and show excellent anti-eosinophilic esophagitis activity.	Cao et al. (2022)
Hybrid membrane-camouflaged ISL nanoparticles (ISL-HM-NPs)	264.59 ± 44.08	Glioma	ISL-HM-NPs increased the solubility of ISL and also enhanced its targeting and antitumor activity.	Shi et al. (2022)
ISL pH-sensitive micelles (ISL-M)	151.15 ± 1.04	Ulcerative colitis	ISL-M increased bioavailability and better prevented colitis by reducing inflammatory.	Shi et al. (2024)
ISL-encapsulated mesoporous silica nanoparticles (MSNs-ISL)	∼200	Lytic bone diseases	MSNs-ISL had anti-osteoclastic effects and prevented bone destruction.	Sun et al. (2019)
iRGD-modified lipid–polymer hybrid nanoparticles loaded with ISL (ISL-iRGD NPs)	138.97 ± 2.44	Breast cancer	ISL-iRGD-NPs can improve anti-breast cancer efficacy and tumor-targeting ability of ISL.	Gao et al. (2017)
ISL-loaded nanostructured lipid carrier (ISL-NLC)	160.73 ± 6.08	Cancer	ISL-NLC had stronger antitumor effect and biodistribution *in vivo* compared to the ISL.	Zhang et al. (2013)
ISL loaded nanoliposomes (ISL-NLs)	48.9 ± 36.2	Colorectal cancer	ISL-NLs regulated AMPK/mTOR mediated glycolysis in colorectal cancer.	Wang et al. (2020a)

Furthermore, the metabolites of ISL have demonstrated potential pharmacological effects and therapeutic benefits for the treatment of a variety of diseases. These metabolites, including liquiritigenin, naringenin chalcone, sulfuretin, butein, and davidigenin (Yang et al., 2016), have been the subject of numerous studies evaluating their individual pharmacological effects, such as neuroprotection, hepatocyte protection, and anti-cancer properties (Kim et al., 2009; Yang et al., 2013; Lu et al., 2024). A detailed summary of the specific pharmacological effects of each metabolite can be found in Table 2.

**TABLE 2:** Summary of the various pharmacological effects of ISL metabolites.

Compound	Structural formula	Pharmacological effects	References
Liquiitigenin	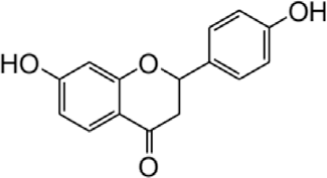	Neuroprotective activities Anti-nociception activities Anti-bacterial activities Anti-asthmatic effects Anti-diabetic activities Hepatoprotective activities Anti-cancer activities	Chokchaisiri et al. (2009), Jayaprakasam et al. (2009), Kim et al. (2009), Straub et al. (2013), Yang et al. (2013), Paterni et al. (2015), Bae et al. (2018)
Naringenin chalcone	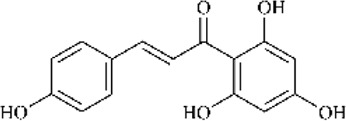	Anti-allergic skin disorders Anti-allergic asthma	Yamamoto et al. (2004), Iwamura et al. (2010), Escribano-Ferrer et al. (2019)
Sulfuretin	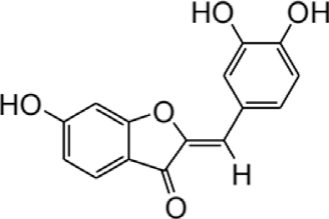	Enhancement of bone growth Anti-depressive effects Hepatoprotective activities Alleviation of atopic dermatitis Prevention of obesity	Jiang and Sun (2018), Kim et al. (2019), Lu et al. (2019), Sun et al. (2022), Chang et al. (2023)
Butein	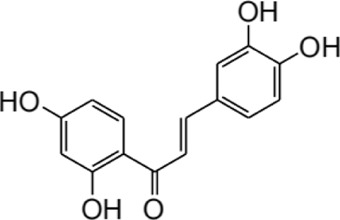	Anti-cancer activities Treatment of bone cancer pain Alleviation of atherosclerosis Treatment of cardiac diseases	Tungalag et al. (2022), Liu et al. (2023), Rehman et al. (2023), Lu et al. (2024)
Davidigenin	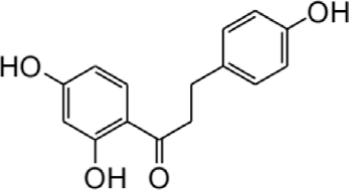	Anti-cancer activities	Liu et al. (2002), Keranmu et al. (2022)

## 6 Limitations and future prospects

All of the above published results suggest that ISL confer protective effects on various disease via Nrf2 signaling pathways. However, due to its low bioavailability and poor delivery characteristics, the big gap between basic research and clinical application still exists. In order to enable ISL available for better therapeutic effects, more trials with ISL either alone or in combination with existing therapies are also needed to fully appreciate its potential. Based on this, the development of ISL formulations may represent a promising avenue for future research. Currently, drug delivery systems such as nanoparticles, liposomes, and micelles has been reported to be applied to enhance drug’s solubility, absorbance, and stability (Zhang et al., 2013; Zhang X. et al., 2016; Xie et al., 2019). Theranostic nanoparticles consisting of ISL and a near-infrared photosensitizer provide a promising delivery platform. It can enhance treatment outcomes, and reduce drug dosage and side effects (Sun et al., 2023). In addition, Studies on the side effects and safety evaluations of this plant are very limited although ISL is widely used in Chinese traditional medicine. In the future, long-term toxicity studies and clinical trials of ISL are still needed.

## 7 Conclusion

Multiple research studies have shown the considerable role of oxidative stress in the development of various illnesses. Consequently, directing therapeutic interventions towards mitigating oxidative stress may offer a pragmatic approach for treating various ailments. Cells possess multiple antioxidant systems that serve to counteract intracellular ROS. Among these systems, the Nrf2/HO-1 signaling pathway represents a prominent endogenous antioxidant mechanism. Nrf2, as the principal regulator of anti-oxidative stress responses, primarily exerts its influence by stimulating the activation of downstream antioxidant and cellular protective genes. As a result, Nrf2 signal pathway activation may inhibit oxidative stress. In this particular context, ISL exhibits both antioxidant characteristics and minimal toxicity as a natural compound. There are numerous studies suggesting that ISL could be used as a novel therapeutic agent for a variety of diseases by modulating the Nrf2 signal pathway in diverse animal and cellular models. In summary, our review provides new insights for ISL as Nrf2 signaling pathway modulator for treating various diseases.

## Glossary

**ACSL4**: Acyl-CoA synthetase long-chain family member 4
**ALF**: acute liver failure
**ALI**: acute lung injury
**AMI**: acute myocardial infarction
**AMPK**: adenosine 5‘-monophosphate (AMP)-activated protein kinase
**ARE**: antioxidant response element
**Bax**: Bcl-2-associated X
**Bcl-2**: B-cell lymphoma-2
**BTB**: Broad complex, Tramtrack and Bric-à-Brac domain
**bZIP**: basic-region leucine zipper
**Caspase-3**: cysteinyl aspartate specific proteinase-3
**CAT**: catalase
**COPD**: chronic obstructive pulmonary disease
**COX-2**: cyclooxygenase-2
**CREB**: cAMP response element-binding protein
**CS**: cigarette smoke
**CTR**: C-terminal region
**Cul3**: cullin3
**DGR**: double glycine repeat domain
**ERK**: extracellular signal-regulated kinase
**FPM**: F127/P123 polymeric micelles
**GBC**: Gallbladder cancer
**GCLC**: glutamate-cysteine ligase catalytic subunit
**GCLM**: glutamate-cysteine ligase modifier subunit
**GPX4**: gluta thione peroxidase 4
**GSH**: glutathione
**GSH-PX**: glutathione peroxidase
**GSK-3β**: glycogen synthase kinase-3β
**GST**: glutathione S-transferase
**HDAC**: histone deacetylase
**HMGB1**: high mobility group box 1 protein
**HMOX1**: haem oxygenase-1
**HO-1**: heme oxygenase-1
**Iba-1**: ionized calcium bindingadaptor molecule-1
**IL-1β**: interleukin-1β
**iNOS**: inducible nitric oxidesynthzase
**ISL**: Isoliquiritigenin
**ISL-HM-NPs**: Hybrid membrane-camouflaged Isoliquiritigenin nanoparticles
**ISL-M**: Isoliquiritigenin pH-sensitive micelles
**MSNs-ISL**: Isoliquiritigenin-encapsulated mesoporous silica nanoparticles
**ISL-iRGD-NPs**: iRGD-modified lipid–polymer hybrid nanoparticles loaded with Isoliquiritigenin
**ISL-NLC**: Isoliquiritigenin-loaded nanostructured lipid carrier
**ISL-NLs**: Isoliquiritigenin loaded nanoliposomes
**IVR**: intervening region
**IκBα**: I-kappa-B-alpha
**JNK**: c-Jun N-terminal kinase
**Keap1**: Kelch-like epichlorohydrin associated protein 1
**LPS**: lipopolysaccharide
**LPS/D-GalN**: LPS/D-galactosamine
**MAPK**: mitogen-activated protein kinase
**MDA**: malondialdehyde
**miRNA**: microRNA
**MPO**: myeloperoxidase
**MRP4**: multidrug resistance protein 4
**NF-κB**: nuclear factor kappa-B
**NLRP3**: NOD-like receptor thermal protein domain associated protein 3
**NPs**: nanocomplexes
**NQO1**: nicotinamide adenine dinucleotide phosphate(NADPH) quinone oxidoreductase-1
**NOX2**: NADPH oxidase 2
**Nrf2**: nuclear factor erythroid-2-related factor 2
**NTR**: N-terminal domain
**PD**: Parkinson’s disease
**PERK**: protein kinase RNA–like endoplasmic reticulum kinase
**PGC-1α**: peroxisome proliferator-activated receptor-gamma coactivator
**PKC**: protein kinase C
**RA**: rheumatoid arthritis
**Rbx1**: RING box protein 1
**ROS**: reactive oxygen species
**SAP**: severe acute pancreatitis
**SCF**: SKP1-CUL1-F-box protein
**SLC7A11**: solute carrier family 7 member 11
**SIRT1**: silent information regulator 1
**SMEDDS**: self-microemulsifying drug delivery system
**SNEDDS**: self-nanoemulsifying drug delivery systems
**SOD**: superoxide dismutase
**SQSTM1**: sequestosome 1
t-BHP: tert-Butyl hydroperoxide
**TBI**: traumatic brain injury
**TNF-α**: tumor necrosis factor-α
**TGF-β**: transforming growth factor-β
**VCAM-1**: vascular cell adhesion molecule-1
**β-TrCP**: β-transducin repeat-containing protein
**4-HNE**: 4-hydroxynonenal
