# Supplementary material for: Isoliquiritigenin as a modulator of the Nrf2 signaling pathway: potential therapeutic implications
Source: Front Pharmacol. 2024 Oct 9;15:1395735. doi: 10.3389/fphar.2024.1395735 (PMC11496173; doi:10.3389/fphar.2024.1395735)
Supplement: Supplementary file 1 [file Table1.docx]

**Supplementary Table 1.** Summary of the most relevant preclinical studies evaluating the therapeutic effects of ISL administration to animals and cells subjected to diverse diseases.

| **Disease** | **Animals** | **Dose range** | **Cell line** | **Dose range** | **Targets and other pathways** | **Outcomes of study** | **References** |
| --- | --- | --- | --- | --- | --- | --- | --- |
| ALI | C57BL/6 mice | ISL (30 mg/kg), intraperitoneally, pretreatment for 1h | RAW 264.7 cells | ISL (5, 10, 20 µM), for 18 h | Nrf2, HO-1, glutamate-cysteine ligase catalytic subunit(GCLC), GCLM, NQO1, AMPK, inducible nitric oxidesynthzase(iNOS), cyclooxygenase-2(COX-2), NOD-like receptor thermal protein domain associated protein 3 (NLRP3), I-kappa-B-alpha(IκBα), p65, GSK-3β, IL-1β | ISL significantly alleviated lung injury, ameliorated inflammatory cell exudation and the production of ROS, and increased the expression of GSH and SOD by activating AMPK/Nrf2/ARE signaling. | (Liu et al., 2017) |
| COPD | C57BL/6N male mice, 6-8 weeks | ISL (10,20,30 mg/kg), orally, 1h before CS administration, twice a day for 4 weeks | —— | —— | p65, IκB, Nrf2, HO-1, MPO, MDA, IL-1β, TNF-α | ISL protected against CS-induced COPD by reducing inflammation and oxidative stress by regulating Nrf2 and NF-κB signaling. | (Yu et al., 2018) |
| Carrageenan-induced pleurisy | Female BALB/c mice | ISL (30 mg/kg), intraperitoneally, twice (each time interval of 12 h) for 24h | —— | —— | Nrf2, Keap1, HO-1, NQO1, GCLC, GCLM, iNOS, COX-2, VCAM-1, JNK, ERK, p38, p65, IκBα, NLRP3, IL-1β, NADPH Oxidase 2(NOX2), NOX4 | ISL exerts protective effects on carrageenan-induced pleurisy and lung injury by exerting antioxidative activities through Nrf2 and anti-inflammatory actions through NLRP3. | (Gao et al., 2020) |
| Doxorubicin-induced hepatic damage | Adult male rats, 10 weeks | ISL (25 mg/kg), orally, given daily but 10 days prior and 10 days after doxorubicin treatment | Alpha mouse liver 12 cells | ISL (10 µM), for 24h prior post-treatment with doxorubicin | MDA, GSH, SOD, IL-6, TNF-α, silent information regulator 1 (SIRT1), NF-κB, Nrf2, B-cell lymphoma-2(Bcl-2), Bcl-2-associated X(Bax), Caspase-3/8/9 | ISL prevented hepatocyte damage, decreased TNF-α, IL-6 as well as Bax levels, and increased GSH levels, SOD, and catalase mainly through stimulating the Nrf2 axis and suppressing NF-κB pathway. | (Al-Qahtani et al., 2022) |
| Triptolide-induced hepatotoxicity | Healthy male ICR mice | ISL (25, 50 mg/kg), orally,  once daily for 7 days | Human normal L02 hepatoc-ytes | ISL (2.5, 5.5, 7.5 µM), for 12h | Nrf2, NQO1, multidrug resistance protein 4 (MRP4), MRP2 | ISL effectively alleviated triptolide-induced hepatotoxicity and reduce the hepatic oxidative stress and hepatic accumulations of both endogenous bile acids and exogenous Triptolide through increasing Nrf2, NQO1, and hepatic influx and efflux transporters expression. | (Hou et al., 2018) |
| Triptolide-induced hepatotoxicity | —— | —— | Human hepatocarcinoma cell line | ISL (5, 10, 20 µM), for 12h | ROS, GSH, Nrf2, HO-1, NQO1, MRP2 | ISL decreased ROS level, enhanced intracellular GSH content, and increased the MRP2, HO-1 and NQO1 expression possibly via activating Nrf2. | (Cao et al., 2016b) |
| Emodin-induced hepatotoxicity | Zebrafish embryo | ISL (1, 2, 4µM), for 72h | Human normal L-02 hepatocytes | ISL (10, 15, 20 µM), for 48h | Nrf2, Bax, Bcl-2, GSH, SOD, MDA, ERK, p38, Keap1, HO-1, NQO1, GST, MRP2, MRP4 | ISL effectively protected EMO-induced liver injury, reduced ROS generation and MDA generation, enhanced the levels of GSH and SOD via activating protein kinases MAPKs and Keap1-Nrf2 signaling pathway. | (Ni et al., 2022) |
| t-BHP-induced  hepatocyte damage | Sprague-Dawley male rats, 6 weeks | ISL (5, 20 mg/kg), orally, for 4 consecutive days | HepG2 cells | ISL (10, 30 µM)), for 12 h | Nrf2, ERK, HO-1, GCLC, NQO1, HO-1 | ISL significantly reduced hepatic damage, inhibited apoptosis, mitochondrial damage and ROS production by activating ERK-mediated Nrf2 pathway. | (Park et al., 2016) |
| Acute liver failure (ALF) | C57BL/6 male mice, 8 weeks | ISL (10, 20, 40 mg/kg), orally, once a day for  3 successive days. | Mouse primary hepatocytes | ISL (5, 10, 20, 40 μM), for 12, 24, and 48 h. | PGC-1α, Nrf2, HO-1, NQO1, Keap1, TNF-α, IL-6, IL-1β, iNOS, NLRP3, Caspase-1/3, Bax, Bcl-2 | ISL improves the ability of anti-oxidative stress, alleviates inflammatory reaction, apoptosis, and inhibits NLRP3 inflammasome to protect (LPS/D-GalN)-induced ALF via the PGC-1α/Nrf2 pathway. | (Wang et al., 2021) |
| Triptolide-induced hepatotoxicity | ICR male  Mice, 6-8 weeks | ISL (12.5, 25, 50mg/kg), daily for 7 days consecutively. | —— | —— | Nrf2, GSH, glutathione peroxidase (GSH-PX), SOD, catalase (CAT), MDA | ISL could protect against triptolide-induced hepatotoxicity, inhibiting MDA and restoring the levels of GSH, GSH-PX, SOD and CAT via activation of the Nrf2 pathway. | (Cao et al., 2016a) |
| Diabetes mellitus-induced  aortic injury | Sprague-Dawley male rats, 6-8 weeks | ISL(20 mg/kg) , orally, for 8 weeks. | —— | —— | Nrf2, Keap1, HO-1, IL-6, IL-10, TNF-α, Caspase-3, MDA, GSH | ISL modulated Nrf2 /Keap1 to restore oxidative/antioxidative stress homeostasis in aorta, suppressed lipid deposition, and restored an almost normal aortic thickness. | (Alzahrani et al., 2021) |
| Ischemia-induced  myocardial injury | C57BL/6J male mice, aged 8-10 weeks | ISL (100 mg/kg), intraperitoneally, for 3 days | —— | —— | Nrf2, HO-1, ROS, MDA, SOD, GSH-Px, HO-1, p65, IκBα, IL-6, IL-1, TNF-α | ISL significantly reduced the myocardial infarction area, enhanced the function of the heart, and alleviated myocardial oxidative stress and inflammation by activating Nrf2 /HO-1 pathway. | (Yao et al., 2022) |
| Diabetic cardiomyopathy | Male C57BL/6 mice, 8 weeks of age | ISL(10, 20mg/kg),  orally, every other day for 12  weeks | Embryonic rat heart-derived H9c2 cells | ISL (2.5, 5, 10, 20, 40 μM), for 24 h | Nrf2, HO-1, NQO1, JNK, p38, ERK, TNF-α, IL-6, IL-1β,  vascular cell adhesion molecule-1(VCAM-1), Collagen-1, Bcl-2, Bax | ISL protected the cardiac function, exhibited anti-inflammatory and antioxidant stress activities via the inhibition of MAPKs and induction of Nrf2 signaling pathway respectively. | (Gu et al., 2020) |
| Acute myocardial-infarction | C57BL6J mice | ISL(100mg/kg), intraperitoneally, pretreatment daily for 3 days | Neonatal mouse cardiomyocytes | ISL(5,25,50μmol/L), for 48h | Nrf2, ACSL4, GPX4, HO-1, 4-hydroxynonenal (4-HNE), solute carrier family 7 member 11(SLC7A11) | ISL reduced myocardial damage through the activation of the Nrf2/HO-1/SLC7A11/GPX4 pathway, which served to inhibit acute myocardial infarction by mitigating oxidative stress, mitochondrial injury, and cardiomyocyte death. | (Yao et al., 2024) |
| Acute pancreatitis | Male ICR mice, 8 weeks of age | ISL (50, 100, 200mg/kg), intraperitoneally, at the beginning of the experiment | —— | —— | Nrf2, HO-1, SOD, MDA, GSH, IL-1β, IL-6 | ISL mitigated the severity of pancreatic tissue injury and pancreatitis-associated lung injury, decreased serum amylase and lipase levels, and reduced oxidative stress injury by modulating the Nrf2 /HO-1 pathway. | (Liu et al., 2018) |
| SAP | Male C57BL/6 mice | —— | —— | —— | Nrf2, HO-1, NQO1, MDA, SOD, IL-6, TNF-α, NF-κB, IκB, Caspase-3 | ISL reduced pancreatic and intestinal injury, repaired the intestinal barrier damage, suppressed oxidative stress and the inflammatory responses via regulation of the Nrf2 /NF-κB pathway. | (Zhang et al., 2018b) |
| Inflammatory bowel disease | —— | —— | HT-29 cell lines | ISL (1, 5, 10, 20 μM), for 1, 3, 6, 12, 24h | IL-8, IL-1β, COX-2, Nrf2 , HO-1, NQO1, high mobility group box 1 protein (HMGB1), histone deacetylase (HDAC), NF-κB, p65, IκBα | ISL exerted Anti-inflammatory effects by suppressing the expression of inflammatory molecules such as HMGB1, IL-8, IL-1β, which may be related with the induction of HDAC activity and induced activation of Nrf2 and expression of its target genes, such as HO-1 and NQO1. | (Chi et al., 2017) |
| Colitis | C57BL/6  mice | ISL (20 mg/kg), orally, treatment for 14 days | —— | —— | Nrf2, NQO1, HO-1, TNF-α, IL-1β, IL-6, GSH-PX, MPO, MDA, SOD | ISL and SFN supplementation regulated oxidative stress and inflammation responses via activating the Keap1–Nrf2 pathway to alleviate colitis. | (Cheng et al., 2022) |
| Hypertensive  renal injury | —— | —— | Human HK-2 proximal tubule epithelial cells | ISL  (2.5, 5, 10 mM), for 24, 48h | Bax, Bcl-2, cleaved Caspase-3, cleaved Caspase-9, SOD, MDA, Nrf2, HO-1, NQO1, p65, IκBα, IL-1β, TNF-α | ISL ameliorated hypertensive renal injury induced by Ang II through suppressing inflammation cytokines, excessive deposition of extracellular matrix and oxidative stress induced apoptosis via Nrf2 and NF-κB pathways. | (Xiong et al., 2018a) |
| Diabetic nephropathy | C57BL/6 mice, 6-8 weeks | ISL(10,20mg/kg), orally, every 2 days for 12 weeks | Rat renal tubular epithelial cell line | ISL (10,20 μM), pre-treatment for 1h | Nrf2, HO-1, p38, ERK, JNK, transforming growth factor-β (TGF-β), Bax, Bcl-2 | ISL prevented diabetic nephropathy, ameliorated the inflammatory reactions and oxidative stress, and regulated p38 MAPK and Nrf2 pathways downstream of SIRT1 . | (Huang et al., 2020) |
| Alzheimer’s disease | —— | —— | BV2 cells and mouse neuroblastoma  (N2a) cells | ISL (1, 5, 10, and 20 µM), for 24 h | IL-1β, IL-6, TNF-α, COX-2, iNOS, Nrf2, HO-1, NQO1, NF-κB | ISL suppresses AβO-induced inflammation and oxidative stress in BV2 cells via regulating the Nrf2 /NF-κB signaling. | (Fu and Jia, 2021) |
| PD | Male C57BL/6J mice | ISL (20mg/kg), intraperitoneally, for 14 days | —— | —— | Iba-1, IL-1β, IL-6, TNF-α, Nrf2, NQO1 | ISL significantly attenuated neurological deficits in PD, reduced neuroinflammation through the activation of the Nrf2 /NQO1 signaling pathway. | (Huang et al., 2022) |
| TBI | Male C57BL/6 mice | ISL (20mg/kg), intraperitoneally, at 1 h post-TBI | SH-SY5Y cells | ISL (20 µM), added 2 h before the cells were placed in an anaerobic chamber for 6 h. | Aquaporin4, cleaved-Caspase-3, GSH-PX, SOD, MDA, HO-1, NQO1 | ISL protect the brain injury against TBI through reducing Garcia neuro score, injury histopathology, cerebral vascular permeability and inhibiting oxidative stress via Nrf2 /HO-1 pathway. | (Zhang et al., 2019b) |
| Kainic acid-induced seizures | Male Wistar rats | ISL (20mg/kg), intraperitoneally, at 30 min, 12 h and 24 h prior to KA injection | —— | —— | cleaved-Caspase-3/9, Nrf2, HO-1, NQO1, NLRP3, TNF-α, IL-1β, IL-18 | ISL protected against cognitive impairment, suppressed synaptic dysfunction, neuronal injury and neuroinflammation via regulating the Nrf2 signaling and the NLRP3 inflammasome pathway. | (Zhu et al., 2019b) |
| Experimental intracerebral  hemorrhage | Adult male Sprague-Dawley rats | ISL (10, 20, 40 mg/kg), intraperitoneally, at 30 min, 12 h, 24 h, and 48 h after ICH induction | —— | —— | NF-κB, p-65, NLRP3, Nrf2, HO-1, NQO1, IL-1β, IL-18, Caspase-1 | ISL reduced early brain impairments and neurological deficits, attenuated the ROS production, regulating the NF-κB on the activation of NLRP3 inflammasome pathway through the triggering of Nrf2 activity and Nrf2 induced antioxidant system. | (Zeng et al., 2017) |
| Experimental diabetic neuropathy | Adult male Sprague-Dawley rats | ISL (10, 20 mg/kg), orally, for 2 weeks | Mouse N2A neuroblastoma cells | ISL (2.5, 5 µM), for 6h | SIRT1, Nrf2, NQO1, PGC-1α, AMPK, Beclin1 | ISL attenuated the experimental diabetic neuropathy injury, reduced oxidative damage and alleviated mitochondrial impairment via SIRT1 activation, facilitating the Nrf2 directed antioxidant signaling. | (Yerra et al., 2017) |
| Viral diseases | Male C57BL/6 mice aged 6-8 weeks | ISL (25, 50 mg/kg/day), intraperitoneally, for 4 days | A549 cells,THP1 cells | ISL (50 μM) was incubated for 1.5h, and then ISL was replaced, for another 8.5h incubation | Nrf2, HO-1, p62, GCLM, SQSTM1 | ISL has antiviral and anti-inflammatory effects in virus infections, which are associated with its ability to activate Nrf2. | (Wang et al., 2023a) |
| Acute myeloid leukemia | —— | —— | Human promyelocytic leukemia cells | ISL (2.5, 5, 10 μg/mL), for 72 h | ROS, MDA, GSH, CAT, GST, GSH-PX, Nrf2, NQO1 | ISL modulate intercellular redox homeostasis and facilitate differentiation as a differentiation-inducing agent possibly via Nrf2 /ARE pathway. | (Chen et al., 2013a) |
| Gallbladder cancer(GBC) | BALB/c nude mice | ISL(50mg/kg), orally, every 2 days for 28 days | Human GBC SGC996 cell line | ISL(30,60,90mmol/L)for24h,48h,72h | Nrf2, Keap1, P62, gluta thione peroxidase 4 (GPX4), Acyl-CoA synthetase long-chain family member 8 (ACSL8), haem oxygenase-1(HMOX1) | ISL triggered ferroptosis in GBC through activation of p62-Keap1-Nrf2-HMOX1 signaling and downregulation of GPX4. | (Wang et al., 2023b) |
| Rheumatoid arthritis(RA) | Sprague-Dawley rats | ISL (10 mg/kg), local injection, injections into the right knee for 3 days | C20A4 human chondrocyte cell line | ISL (3,5,10 μM), pretreated for 1 h | HO-1, Bax, Bcl-2, Caspase-3, iNOS, PGC-1α, Nrf2, SIRT1 | ISL exerted anti-oxidation, anti-inflammation, anti-apoptosis through activation Nrf2/HO-1 pathway in RA. | (Hung et al., 2024) |
| Chronic prostatitis and chronic pelvic pain syndrome | Nonobese diabetic mice | ISL(50,100,200mg/kg), intraperitoneally, treatment started two days before the subsequent immunization | Mouse macrophage RAW 264.7 cells | ISL (20μmol/ml), for 4 h | Nrf2, HO-1, NLRP3, Caspase -1, IL-1β, Nrf2, SOD, MDA | ISL attenuated experimental autoimmune prostatitis through activation Nrf2 and downregulating the NLRP3 inflammasome pathway. | (Feng et al., 2024) |

**Referrences**

Al-Qahtani, W.H., Alshammari, G.M., Ajarem, J.S., Al-Zahrani, A.Y., Alzuwaydi, A., Eid, R., and Yahya, M.A. (2022). Isoliquiritigenin prevents Doxorubicin-induced hepatic damage in rats by upregulating and activating SIRT1. *Biomed Pharmacother* 146**,** 112594.

Alzahrani, S., Said, E., Ajwah, S.M., Alsharif, S.Y., El-Bayoumi, K.S., Zaitone, S.A., Qushawy, M., and Elsherbiny, N.M. (2021). Isoliquiritigenin attenuates inflammation and modulates Nrf2/caspase-3 signalling in STZ-induced aortic injury. *J Pharm Pharmacol* 73**,** 193-205.

Cao, L., Yan, M., Ma, Y.X., Zhang, B.K., Fang, P.F., Xiang, D.X., Li, Z.H., Gong, H., Deng, Y., and Li, H.D. (2016a). Isoliquiritigenin protects against triptolide-induced hepatotoxicity in mice through Nrf2 activation. *Pharmazie* 71**,** 394-397.

Cao, L.J., Li, H.D., Yan, M., Li, Z.H., Gong, H., Jiang, P., Deng, Y., Fang, P.F., and Zhang, B.K. (2016b). The Protective Effects of Isoliquiritigenin and Glycyrrhetinic Acid against Triptolide-Induced Oxidative Stress in HepG2 Cells Involve Nrf2 Activation. *Evid Based Complement Alternat Med* 2016**,** 8912184.

Chen, H., Zhang, B., Yuan, X., Yao, Y., Zhao, H., Sun, X., and Zheng, Q. (2013a). Isoliquiritigenin-induced effects on Nrf2 mediated antioxidant defence in the HL-60 cell monocytic differentiation. *Cell Biol Int* 37**,** 1215-1224.

Cheng, X.R., Yu, B.T., Song, J., Ma, J.H., Chen, Y.Y., Zhang, C.X., Tu, P.H., Muskat, M.N., and Zhu, Z.G. (2022). The Alleviation of Dextran Sulfate Sodium (DSS)-Induced Colitis Correlate with the logP Values of Food-Derived Electrophilic Compounds. *Antioxidants (Basel)* 11.

Chi, J.H., Seo, G.S., Cheon, J.H., and Lee, S.H. (2017). Isoliquiritigenin inhibits TNF-α-induced release of high-mobility group box 1 through activation of HDAC in human intestinal epithelial HT-29 cells. *Eur J Pharmacol* 796**,** 101-109.

Feng, R., Meng, T., Zhao, X., Yu, W., Li, H., Wang, Z., Chen, J., and Yang, C. (2024). Isoliquiritigenin reduces experimental autoimmune prostatitis by facilitating Nrf2 activation and suppressing the NLRP3 inflammasome pathway. *Mol Immunol* 169**,** 37-49.

Fu, Y., and Jia, J. (2021). Isoliquiritigenin Confers Neuroprotection and Alleviates Amyloid-β42-Induced Neuroinflammation in Microglia by Regulating the Nrf2/NF-κB Signaling. *Front Neurosci* 15**,** 638772.

Gao, Y., Lv, X., Yang, H., Peng, L., and Ci, X. (2020). Isoliquiritigenin exerts antioxidative and anti-inflammatory effects via activating the KEAP-1/Nrf2 pathway and inhibiting the NF-κB and NLRP3 pathways in carrageenan-induced pleurisy. *Food Funct* 11**,** 2522-2534.

Gu, X., Shi, Y., Chen, X., Sun, Z., Luo, W., Hu, X., Jin, G., You, S., Qian, Y., Wu, W., Liang, G., Wu, G., Chen, Z., and Chen, X. (2020). Isoliquiritigenin attenuates diabetic cardiomyopathy via inhibition of hyperglycemia-induced inflammatory response and oxidative stress. *Phytomedicine* 78**,** 153319.

Hou, Z., Chen, L., Fang, P., Cai, H., Tang, H., Peng, Y., Deng, Y., Cao, L., Li, H., Zhang, B., and Yan, M. (2018). Mechanisms of Triptolide-Induced Hepatotoxicity and Protective Effect of Combined Use of Isoliquiritigenin: Possible Roles of Nrf2 and Hepatic Transporters. *Front Pharmacol* 9**,** 226.

Huang, L., Han, Y., Zhou, Q., Sun, Z., and Yan, J. (2022). Isoliquiritigenin attenuates neuroinflammation in mice model of Parkinson's disease by promoting Nrf2/NQO-1 pathway. *Transl Neurosci* 13**,** 301-308.

Huang, X., Shi, Y., Chen, H., Le, R., Gong, X., Xu, K., Zhu, Q., Shen, F., Chen, Z., Gu, X., Chen, X., and Chen, X. (2020). Isoliquiritigenin prevents hyperglycemia-induced renal injuries by inhibiting inflammation and oxidative stress via SIRT1-dependent mechanism. *Cell Death Dis* 11**,** 1040.

Hung, S.Y., Chen, J.L., Tu, Y.K., Tsai, H.Y., Lu, P.H., Jou, I.M., Mbuyisa, L., and Lin, M.W. (2024). Isoliquiritigenin inhibits apoptosis and ameliorates oxidative stress in rheumatoid arthritis chondrocytes through the Nrf2/HO-1-mediated pathway. *Biomed Pharmacother* 170**,** 116006.

Liu, Q., Lv, H., Wen, Z., Ci, X., and Peng, L. (2017). Isoliquiritigenin Activates Nuclear Factor Erythroid-2 Related Factor 2 to Suppress the NOD-Like Receptor Protein 3 Inflammasome and Inhibits the NF-κB Pathway in Macrophages and in Acute Lung Injury. *Front Immunol* 8**,** 1518.

Liu, X., Zhu, Q., Zhang, M., Yin, T., Xu, R., Xiao, W., Wu, J., Deng, B., Gao, X., Gong, W., Lu, G., and Ding, Y. (2018). Isoliquiritigenin Ameliorates Acute Pancreatitis in Mice via Inhibition of Oxidative Stress and Modulation of the Nrf2/HO-1 Pathway. *Oxid Med Cell Longev* 2018**,** 7161592.

Ni, B., Liu, Y., Gao, X., Cai, M., Fu, J., Yin, X., Ni, J., and Dong, X. (2022). Isoliquiritigenin attenuates emodin-induced hepatotoxicity in vivo and in vitro through Nrf2 pathway. *Comp Biochem Physiol C Toxicol Pharmacol* 261**,** 109430.

Park, S.M., Lee, J.R., Ku, S.K., Cho, I.J., Byun, S.H., Kim, S.C., Park, S.J., and Kim, Y.W. (2016). Isoliquiritigenin in licorice functions as a hepatic protectant by induction of antioxidant genes through extracellular signal-regulated kinase-mediated NF-E2-related factor-2 signaling pathway. *Eur J Nutr* 55**,** 2431-2444.

Wang, H., Jia, X., Zhang, M., Cheng, C., Liang, X., Wang, X., Xie, F., Wang, J., Yu, Y., He, Y., Dong, Q., Wang, Y., and Xu, A. (2023a). Isoliquiritigenin inhibits virus replication and virus-mediated inflammation via NRF2 signaling. *Phytomedicine* 114**,** 154786.

Wang, L., Wang, X., Kong, L., Wang, S., Huang, K., Wu, J., Wang, C., Sun, H., Liu, K., and Meng, Q. (2021). Isoliquiritigenin alleviates LPS/ D-GalN-induced acute liver failure by activating the PGC-1α/ Nrf2 pathway to reduce oxidative stress and inflammatory response. *Int Immunopharmacol* 100**,** 108159.

Wang, Z., Li, W., Wang, X., Zhu, Q., Liu, L., Qiu, S., Zou, L., Liu, K., Li, G., Miao, H., Yang, Y., Jiang, C., Liu, Y., Shao, R., Wang, X., and Liu, Y. (2023b). Isoliquiritigenin induces HMOX1 and GPX4-mediated ferroptosis in gallbladder cancer cells. *Chin Med J (Engl)* 136**,** 2210-2220.

Xiong, D., Hu, W., Ye, S.T., and Tan, Y.S. (2018a). Isoliquiritigenin alleviated the Ang II-induced hypertensive renal injury through suppressing inflammation cytokines and oxidative stress-induced apoptosis via Nrf2 and NF-κB pathways. *Biochem Biophys Res Commun* 506**,** 161-168.

Yao, D., Bao, L., Wang, S., Tan, M., Xu, Y., Wu, T., Zhang, Z., and Gong, K. (2024). Isoliquiritigenin alleviates myocardial ischemia-reperfusion injury by regulating the Nrf2/HO-1/SLC7a11/GPX4 axis in mice. *Free Radic Biol Med* 221**,** 1-12.

Yao, D., Shi, B., Wang, S., Bao, L., Tan, M., Shen, H., Zhang, Z., Pan, X., Yang, Y., Wu, Y., and Gong, K. (2022). Isoliquiritigenin Ameliorates Ischemia-Induced Myocardial Injury via Modulating the Nrf2/HO-1 Pathway in Mice. *Drug Des Devel Ther* 16**,** 1273-1287.

Yerra, V.G., Kalvala, A.K., and Kumar, A. (2017). Isoliquiritigenin reduces oxidative damage and alleviates mitochondrial impairment by SIRT1 activation in experimental diabetic neuropathy. *J Nutr Biochem* 47**,** 41-52.

Yu, D., Liu, X., Zhang, G., Ming, Z., and Wang, T. (2018). Isoliquiritigenin Inhibits Cigarette Smoke-Induced COPD by Attenuating Inflammation and Oxidative Stress via the Regulation of the Nrf2 and NF-κB Signaling Pathways. *Front Pharmacol* 9**,** 1001.

Zeng, J., Chen, Y., Ding, R., Feng, L., Fu, Z., Yang, S., Deng, X., Xie, Z., and Zheng, S. (2017). Isoliquiritigenin alleviates early brain injury after experimental intracerebral hemorrhage via suppressing ROS- and/or NF-κB-mediated NLRP3 inflammasome activation by promoting Nrf2 antioxidant pathway. *J Neuroinflammation* 14**,** 119.

Zhang, M., Huang, L.L., Teng, C.H., Wu, F.F., Ge, L.Y., Shi, Y.J., He, Z.L., Liu, L., Jiang, C.J., Hou, R.N., Xiao, J., Zhang, H.Y., and Chen, D.Q. (2019b). Correction to: Isoliquiritigenin Provides Protection and Attenuates Oxidative Stress-Induced Injuries via the Nrf2-ARE Signaling Pathway After Traumatic Brain Injury. *Neurochem Res* 44**,** 510-511.

Zhang, M., Wu, Y.Q., Xie, L., Wu, J., Xu, K., Xiao, J., and Chen, D.Q. (2018b). Isoliquiritigenin Protects Against Pancreatic Injury and Intestinal Dysfunction After Severe Acute Pancreatitis via Nrf2 Signaling. *Front Pharmacol* 9**,** 936.

Zhu, X., Liu, J., Huang, S., Zhu, W., Wang, Y., Chen, O., and Xue, J. (2019b). Neuroprotective effects of isoliquiritigenin against cognitive impairment via suppression of synaptic dysfunction, neuronal injury, and neuroinflammation in rats with kainic acid-induced seizures. *Int Immunopharmacol* 72**,** 358-366.
